# Effects of Sensory Behavioral Tasks on Pain Threshold and Cortical Excitability

**DOI:** 10.1371/journal.pone.0052968

**Published:** 2013-01-03

**Authors:** Magdalena Sarah Volz, Vanessa Suarez-Contreras, Mariana E. Mendonca, Fernando Santos Pinheiro, Lotfi B. Merabet, Felipe Fregni

**Affiliations:** 1 Laboratory of Neuromodulation, Department of Physical Medicine and Rehabilitation, Spaulding Rehabilitation Hospital and Massachusetts General Hospital, Harvard Medical School, Boston, Massachusetts, United States of America; 2 Charité Center for Neurology, Neurosurgery and Psychiatry, Campus Charité Mitte, Charité - Universitätsmedizin Berlin, Berlin, Germany; 3 Department of Ophthalmology, Massachusetts Eye and Ear Infirmary, Harvard Medical School, Boston, Massachusetts, United States of America; Hospital Nacional de Parapléjicos, Spain

## Abstract

**Background/Objective:**

Transcutaneous electrical stimulation has been proven to modulate nervous system activity, leading to changes in pain perception, via the peripheral sensory system, in a bottom up approach. We tested whether different sensory behavioral tasks induce significant effects in pain processing and whether these changes correlate with cortical plasticity.

**Methodology/Principal Findings:**

This randomized parallel designed experiment included forty healthy right-handed males. Three different somatosensory tasks, including learning tasks with and without visual feedback and simple somatosensory input, were tested on pressure pain threshold and motor cortex excitability using transcranial magnetic stimulation (TMS). Sensory tasks induced hand-specific pain modulation effects. They increased pain thresholds of the left hand (which was the target to the sensory tasks) and decreased them in the right hand. TMS showed that somatosensory input decreased cortical excitability, as indexed by reduced MEP amplitudes and increased SICI. Although somatosensory tasks similarly altered pain thresholds and cortical excitability, there was no significant correlation between these variables and only the visual feedback task showed significant somatosensory learning.

**Conclusions/Significance:**

Lack of correlation between cortical excitability and pain thresholds and lack of differential effects across tasks, but significant changes in pain thresholds suggest that analgesic effects of somatosensory tasks are not primarily associated with motor cortical neural mechanisms, thus, suggesting that subcortical neural circuits and/or spinal cord are involved with the observed effects. Identifying the neural mechanisms of somatosensory stimulation on pain may open novel possibilities for combining different targeted therapies for pain control.

## Introduction

Pain perception can be influenced by several factors such as expectation, drugs, attention and emotional state. In this context methods to induce changes in the pain-related neural network can alter pain perception. The somatosensory system has been a traditional target for modulation of pain perception. Transcutaneous electrical stimulation (TENS) is an example of an intervention that targets the peripheral sensory system leading to changes in pain perception. Pain modulation with TENS is hypothesized to induce effects at spinal and also thalamic levels [1]. Although extensive data from TENS studies have confirmed the notion that modulation of somatosensory system at peripheral level leads to a change in pain perception, one important question is whether behavioral tasks involved with somatosensory processing would have significant effects in pain processing.

It is known that the sensory system is functionally and structurally connected to the motor system. This is why treatments aiming at increasing motor control can be used for pain control [2], [3]. Moreover, recent data has shown that also cortical structures such as the primary motor cortex (M1) can reduce pain significantly [4]. This raises another question, whether sensory behavioral tasks would have an effect on motor cortex plasticity. Moreover, Ostry et al. showed that also learning tasks with motor component are not only related to changes in motor areas but also in brain areas that mediate sensory changes, including primary (S1) and secondary (S2) somatosensory cortices [5]. This is likely mediated by ipsilateral corticocortical pathways connecting motor and somatosensory areas [6], [7]. Furthermore, recent studies showed that the sensory-discriminative dimension of pain is related to pain processing and its modulation in S1 and S2 cortices [8], [9], [10], [11], [12], which project through the lateral thalamic nuclei and other brain areas forming the lateral pain system [13]. Therefore, we aimed to explore if somatosensory input can modify pressure pain thresholds through modulation of sensory and/or motor cortical areas, which can secondarily lead to inhibition of other structures mediating pain processing such as thalamus. Furthermore, we hypothesized that they may change S1 and M1 excitability, respectively, as they require sensory-motor integration.

We therefore measured three tasks involved with somatosensory activation. In two of them we aimed to induce a significant component involved with cortical processing as tasks were associated with somatosensory learning with and without visual feedback [14], [15] while in the third task there was only passive activation of the somatosensory system. In addition we measured motor cortex plasticity as the motor cortex is an important target involved in pain control. In fact, M1 appears to be the best cortical target for pain control when using neuromodulatory techniques [16], [17], [18], [19]. However, the underlying mechanisms of the analgesic effects remain unknown. On one hand, there is recent evidence showing that changes in pain perception and changes in motor-cortical excitability are dissociated [20]. But on the other hand, there is the idea that the altered activity in M1 might be a marker of chronic pain. The study by Lefaucheur et al. (2006) has shown that chronic neuropathic pain is correlated with changes in motor cortex excitability, particularly a decreased inhibition, suggesting impaired GABAergic neurotransmission [21]. Therefore, the aim of our study was to investigate the effects of different somatosensory tasks on pain perception and motor cortex excitability in healthy male individuals as to compare tasks involved with and without cortical processing vs. a control condition. For that reason, the local vs. distant effects were measured in this study: pressure pain threshold in both hands and neurophysiologic measures of cortical plasticity as assessed by transcranial magnetic stimulation (TMS).

Our initial hypothesis is that somatosensory tasks are capable of changing both pressure pain threshold and motor-cortical excitability.

## Methods

### Study Design

We conducted a randomized parallel designed trial to determine the effects of three different somatosensory tasks on pressure pain threshold and M1 excitability in healthy male volunteers. This study was approved by the local ethics review board of Spaulding Rehabilitation Hospital (Boston, USA) and was carried out according to the tenants of the Declaration of Helsinki. Subjects read and signed written informed consent form before participating in this study.

### Subjects

Forty healthy right-handed male subjects (mean age: 25.9 years, SD: 7.79, range: 18–48 years) were recruited by postings in universities, the internet and public places around the Boston area. Subjects were enrolled in the study if they fulfilled the following criteria: (1) adult males; (2) right-handed (confirmed by Edinburgh Handedness Inventory [22]); (3) no use of central nervous system-effective medication; (4) no clinically significant or unstable medical, neurological or psychiatric disorder (assessed with Beck Depression Inventory [23]); (5) no rheumatologic disease (6); no history of alcohol or substance abuse within the last 6 months; and (7) no contraindication to TMS [24]. All subjects gave written, informed consent. Since it is known that high age and the menstrual cycle modulates cortical excitability [25], [26], we enrolled only adult male participants (age range in the study varied from 18 to 48 years old). Moreover, we only included right-handed subjects in order to test the right non-dominant hemisphere. The non-dominant site was chosen for several reasons: firstly, we assumed that the effects of somatosensory learning may be larger for the non-dominant hemisphere as this is known for motor tasks (thus we aimed to avoid ceiling effect), secondly, we thought that the analgesic effects would be greater in the non-dominant hand since it appears to more sensitive to stimuli as compared to the dominant hand.

### Experiment

Subjects were randomized into one of four study arms; thus, each intervention was completed by ten subjects. Every visit included the same assessments and only the 20-minute task - which was performed with subjects’ left hand - was different (SL_sighted_, SL_blindfold_, S_activation_, control, see below). Pressure pain threshold levels were assessed for both hands before and after the task, as well as TMS measures. The investigator assessing the outcomes was blinded to the intervention and although subjects were not blinded as the tasks consisted on different behavioral interventions (not possible to blind), they were not told whether any specific task would be associated with an effect on pain threshold and/or cortical excitability. The left non- dominant hand received the sensory tasks, which means that the task targeted that hand, whereas the right hand was not targeted by the intervention and did not receive any sensory stimulation.

### Somatosensory Tasks

All subjects were seated in the same chair and asked to position their left hand on a table supported with a towel underneath to avoid an uncomfortable arm position. Subjects were instructed not to move their fingers or wrists. In case a participant moved three or more times, which was visually monitored by an investigator, their data were excluded from analyses to control for a pure effect due to somatosensory activation. In total, there were ten subjects randomized in each of the following groups:

#### Somatosensory Learning with visual feedback (SL_sighted_)

This tactile pattern discrimination task consisted of recognizing embossed raised dot patterns. Similar tasks have been described before [15], [27]. The tactile patterns used in the task corresponded to Braille print symbols (produced with a Braille embosser). The recognition of these tactile patterns required sensory and spatial integration [28]. The tactile task was subdivided into four 5 min blocks. Before each block, subjects could view and memorize the pattern and its corresponding name. The sheets with raised patterns were then swept underneath the subject’s left finger in a standardized vertical movement (by an experienced experimenter) keeping the same speed throughout the task. Participants had to name each letter, before the next letter was presented. Furthermore, they were not able to view the shape of the patterns, which was ensured using a blind, however subjects kept visual feedback. To promote continuous learning effects, task difficulty was increased over time by adding patterns of greater complexity. Somatosensory learning (SSL) component was measured by comparing the number of correct letters of the first and last block, which included the same battery of letters.

#### Somatosensory Learning without visual feedback (SL_blindfold_)

This task involved exactly the same procedure as SL_sighted_. The only difference was that the subject wore a specially designed blindfold to ensure no visual input.

#### Simple somatosensory activation (S_activation_)

Participants’ left index finger was excited with items of different texture to activate the somatosensory cortex without the involvement of a learning component. S1 activation and attention was secured by alteration of different textures. Participants were instructed to concentrate on the stimulated finger in order to ensure the same level of vigilance as it was during the learning tasks.

#### Control

The subjects in this group were positioned similarly to the other groups; however, they did not receive any somatosensory input (SSI). Moreover, they were instructed not to talk and to remain attentive, and not to move the hand or finger to exactly mimic the testing conditions of the other tasks. It was visually monitored that participants had a uniform level of attention compared to the other tasks in order to secure comparability of experimental setting.

### Pain Assessment

Pressure pain thresholds were evaluated with an algometer device (model commander, J Tech Medical Industries, USA). This device has a 1-cm^2^ rubber probe, which was pressed against the palmar side of the hand. Subjects were instructed to signal when the stimulus became painful [29]. Threshold levels were assessed before and after the intervention, and the average of three measures were calculated.

### Transcranial Magnetic Stimulation (TMS)

TMS was assessed before and after the intervention. It was performed using a Bistim2 stimulator and a figure-of-eight coil (Magstim Company LTDA, UK). Silver/silver chloride electrodes were placed over the first dorsal interosseus (FDI) muscle belly and its corresponding tendon on the distal phalanx of the index finger. Motor-evoked potentials (MEP) were processed through Powerlab 4/30 (ADinstruments, USA) with a band pass of 20–2000 kHz. Recordings were saved on a computer and off-line analyses were performed with data collection software LabChart (ADinstruments, USA).

TMS assessments were performed on the right hemisphere before and after the intervention. Responses to stimuli were recorded from the contralateral FDI. First, motor threshold (MT) was determined, which was defined as the lowest intensity eliciting 3 out of 5 MEP with an amplitude of 100 µV. MEP were recorded at an intensity that could elicit a MEP of 1 mV (peak-to-peak amplitude). Furthermore, single-pulse measures included cortical silent periods (CSP) at intensities of 110%, 120% and 130% of the MT. During recording, subjects were instructed to perform isometric voluntary contraction with approximately 10% of maximal contraction as ascertained and controlled with a mechanical pinch gauge (Baseline® Evaluation Instruments). Thirty CSP were elicited in a random order. Relative CSP were analyzed, i.e. the entire duration of the last MEP followed by the silent period. Paired-pulse measures included short intracortical inhibition (SICI) with interstimulus interval (ISI) of 3 ms, and intracortical facilitation (ICF) with ISI of 10 ms. The first subthreshold stimulus was set at 70% of the individual MT and the second suprathreshold stimulus at MEP intensity. Forty-five recordings were made in random order having an interval of approximately 8 seconds between each pulse. Paired-pulse measures were analyzed calculating their individual index (SICI or ICF/MEP).

### Statistical Analyses

Analyses were done with STATA (v11.0, College Station, Texas, US) and graphs were generated by GraphPad Prism version 4.00 for Windows (GraphPad Software, USA).

To assess the learning component of the two tasks SL_sighted_ and SL_blindfold_, ANOVA was performed. Moreover, post hoc paired two-tailed t-tests were done separately for each task (SL_sighted_ and SL_blindfold_) to compare the results of the first and last sequence of the tactile task. Thus, these analyses can reveal the change of performance over time and can detect a potential learning experience.

For the main outcome of pressure pain threshold levels, several models of mixed ANOVA were performed as to investigate the effects of hand, time, task and the interaction of time and hand. Subsequently we tested the results of each hand separately and also compared the effects of active tasks (SL_sighted_, SL_blindfold_, S_activation_) together vs. control and separate effects of the sensory tasks. When appropriate, post hoc analyses were done using paired and unpaired two-tailed t-tests to compare changes in pain thresholds within (against baseline) and between groups. In detail we did following analyses: (i) We conducted an ANOVA model to reveal the effects for the interaction of hand and time, (ii) we then conducted separate ANOVAs for the left and right hands comparing active vs. control tasks, (iii) we then ran an ANOVA to test whether the three active sensory tasks (SL_sighted_, SL_blindfold_, S_activation_) induced differential effects compared to controls.

To analyze TMS data, we performed a mixed ANOVA model in which the dependent variables were the measures of cortical excitability (MTs, MEPs, SICIs, ICFs, CSPs) and the independent variables were groups (SL_sighted_, SL_blindfold_, S_activation_, versus Control), time (pre, post) and the interaction of group and time. Moreover, we performed post hoc t-tests to reveal differences within (against baseline) and between groups. Firstly, we conducted an ANOVA for the interaction between task and time comparing somatosensory tasks vs. control. To reveal the direction of changes in cortical plasticity (increase or decrease), we ran post hoc t-tests for all sensory tasks and controls comparing the value before vs. after the intervention. After that, we conducted an ANOVA to reveal potential differences across the three sensory tasks.

Statistical significance and trend refer to a p-value <0.05 and <0.1, respectively.

## Results

All 40 enrolled subjects completed the study and no adverse effects were experienced throughout the study. No participants’ data had to be excluded from the analyses since non of the subjects moved fingers or wrists more then three times during the tasks.

### Behavioral Results: Somatosensory Learning

**Figure 1:**
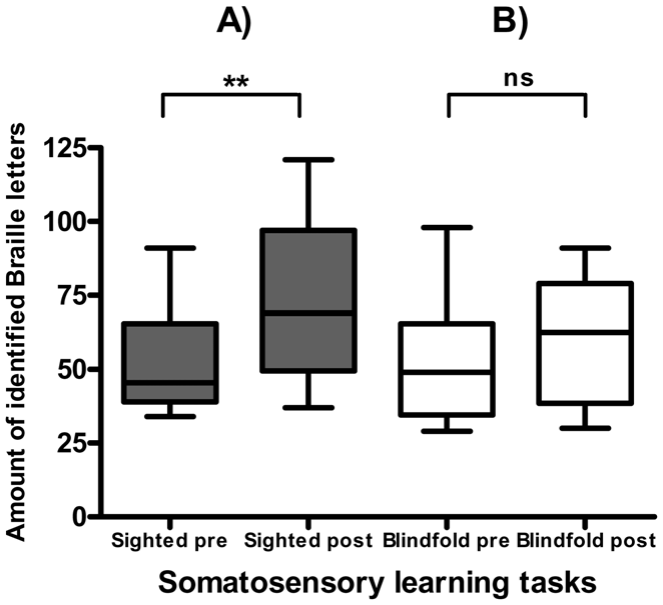
Results of somatosensory learning tasks. A) Results of SL_sighted_. B) Result of SL_blindfold_. Axis of ordinates shows amount of identified tactile patterns; axis of abscissae shows time (first and last block). ** = p<0.01. Ns = not significant.

ANOVA for learning results showed no significant main effect of group (F_(1,19)_ = 0.29, p = 0.5987); however there was a significant effect for the factor time (F_(1,19)_ = 10.76, p = 0.0039) and the interaction of task vs. time (F_(2,18)_ = 6.52, p = 0.0074). Post-hoc analysis for SL_sighted_ comparing the amount of identified tactile patterns of the first vs. last sequence showed a significant result (p = 0.0055); for errors, there were no significant changes (p = 0.5814). In contrast, SL_blindfold_ did not reach significant level for both the correct sequences (p = 0.2303) and the amount of errors (p = 0.3859). This indicates that successful learning occurred only in SL_sighted_ (Figure 1).

### Pain Threshold

We conducted this analysis to respond to three questions: (i) was there a difference in pain threshold changes (before vs. after task) when comparing left vs. right hands? (ii) was there a difference in pain threshold between active sensory tasks vs. control task? and (iii) was there a difference in pain threshold changes between active tasks?

To respond question (i) we conducted an ANOVA model that revealed significant effects for the interaction of hand and time (F_(1,117)_ = 18.71, p = 0.00001). This indicates that the effects of the experiment over time were different between both hands. In fact, in the left hand, there was a significant increase in pain threshold of 1.14 (±1.75) (baseline was 6.38±2.77) (p = 0.00126), and in the right hand there was a significant decrease in pain threshold of 0.84 (±1.2) (baseline was 8.16±3.08) (p = 0.00059).

**Figure 2:**
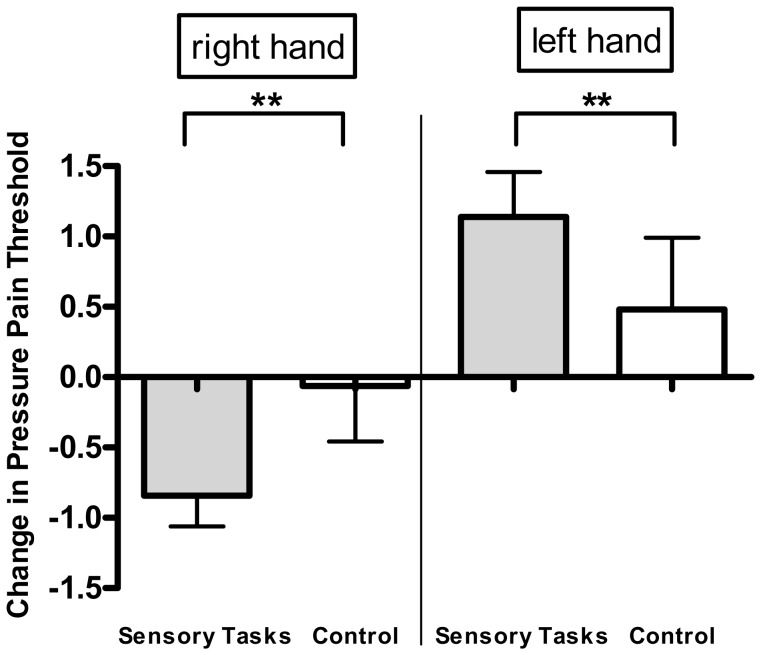
Changes in pressure pain threshold of somatosensory tasks and controls for the right and left hand. ** = p<0.01 as revealed by ANOVA models for the interaction between task and time separately for each hand. Errors bars show standard error of the mean.

**Figure 3:**
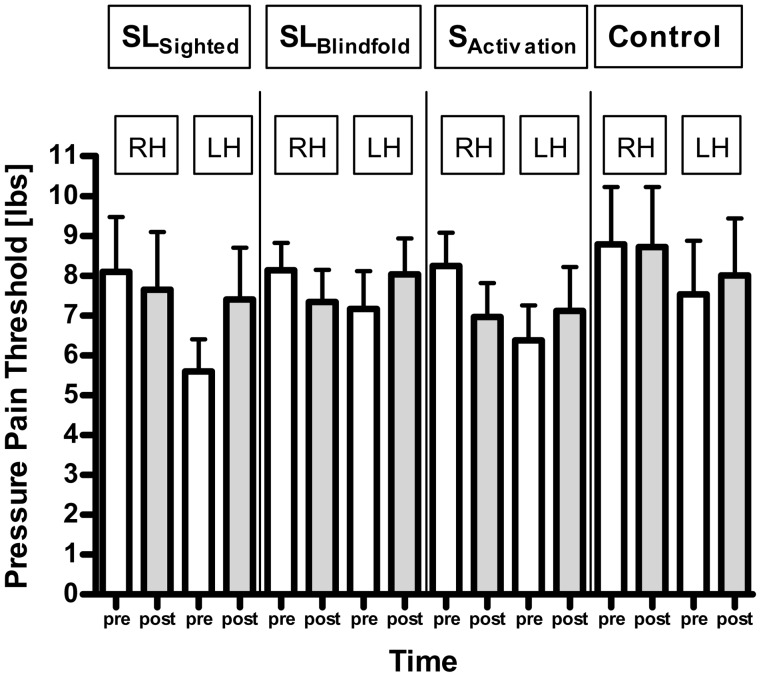
Pressure pain threshold levels. Pressure pain threshold levels before and after the interventions of the left (target to sensory tasks) and right hand for all four study groups (SL_sighted_, SL_blindfold_, S_activation_, control). Errors bars show standard error of the mean. RT: right hand. LT: left hand.

To respond question (ii), we conducted separate ANOVAs for the left and right hands comparing active vs. control tasks. ANOVA models showed significant interaction between task and time for the left (F_(2,38)_ = 7.00, p = 0.0026) as well as the right (F_(2,38)_ = 7.29, p = 0.0021) hand, indicating that the tasks changed pain perception over time differently then the control group (Figure 2). Furthermore ANOVA models of the three sensory tasks only showed significant time effects for each hand (left hand: F_(1,29)_ = 12.75, p = 0.0013; right hand: F_(1,29)_ = 14.88, 0.0006), whereas the control group did not have significant changes (F_(1,9)_ = 0.89, left: p = 0.37; F_(1,9)_ = 0.03, right: p = 0.88) (Figure 3).

To answer question (iii) we used the same model as to address question (ii); but we separated the active tasks (SL_sighted_, SL_blindfold_, S_activation_) vs. control. The results showed significant interaction time vs. group for both left (F_(4,36)_ = 4.10, p = 0.0077) and right (F_(4,36)_ = 4.30, p = 0.0060) hands. Then we performed the same ANOVA without the control group, thus, compared only three sensory tasks across each other. The results showed no significant effect of interaction task vs. time for left hand (F_(2,27)_ = 1.01, p = 0.35) as well as for the right hand (F_(2,27)_ = 1.26, p = 0.03) when considering only the sensory tasks, indicating that three active tasks had similar effect on pain threshold.

### Transcranial Magnetic Stimulation (TMS)

**Figure 4:**
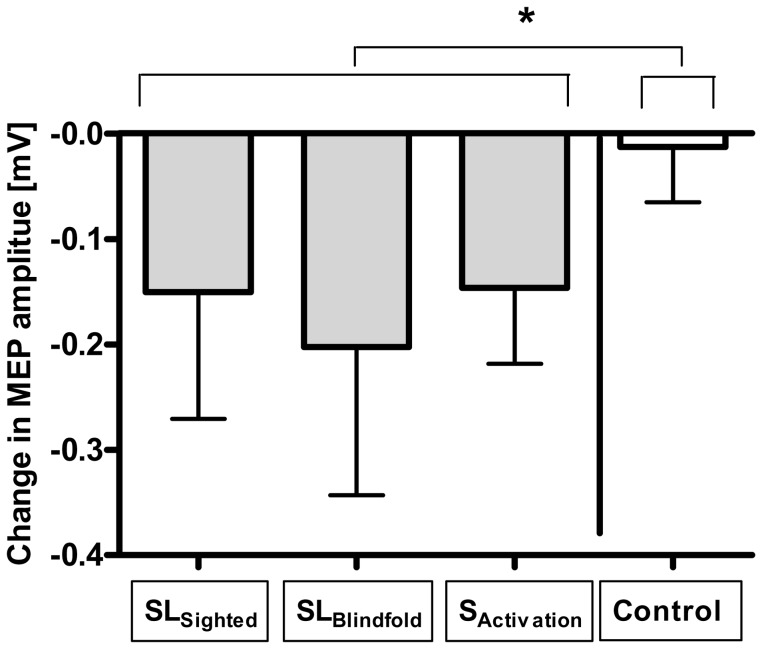
Changes in motor-evoked potentials. Motor-evoked potentials (MEP in mV) for all three somatosensory tasks separately and controls. * = p<0.05 as revealed by ANOVA for the interaction between task and time. Errors bars show standard error of the mean.

For TMS results (Table 1), we conducted similar analyses; however as only the left hand was tested, this analysis was limited to left hand. Initially we analysed whether there was a change of cortical excitability measurements (before vs. after intervention) when comparing somatosensory tasks vs. control. ANOVA showed a significant interaction between task and time for MEP (amplitude: F_(2,38)_ = 4.14, p = 0.0236) and SICI (amplitude: F_(1,35)_ = 6.37, p = 0.016), indicating that somatosensory tasks changed cortical excitability over time differently compared to the control tasks (Figure 4). Post-hoc analyses with all sensory tasks comparing before vs. after the intervention revealed a significant decrease in cortical excitability for MEP (amplitude: p = 0.015; before: 1.58 mV ±0.57; after: 1.41 mV ±0.58). For SICI, although the comparison against baseline was not significant, there was a significant difference when comparing changes in SICI between sensory tasks vs. control (p = 0.037); showing that SICI tend to increase in the sensory tasks group.

**Table 1:** Results of the transcranial magnetic stimulation measurements.

	Transcranial Magnetic Stimulation Measurements								
Task	MEP amplitude	MEP integral	ICF amplitude	ICF integral	SICI amplitude	SICI integral	CSP 110%	CSP 120%	CSP 130%
**SL_sighted_**	1.74 [±0.78]	34.52 [±17.60]	0.271 [±0.042]	0.136 [±0.05]	0.069 [±0.07]	0.021 [±0.023]	68.24 [±18.80]	92.62 [±26.07]	114.9 [±32.43]
	1.59 [±0.73]	28.81 [±13.91]	0.264 [±0.098]	0.134 [±0.03]	0.056 [±0.042]	0.016 [±0.014]	73.47 [±20.67]	95.65 [±30.02]	116.6 [±32.56]
**SL_blindfold_**	1.67 [±0.50]	31.30 [±11.24]	0.356 [±0.246]	0.284 [±0.26]	0.137 [±0.25]	0.100 [±0.193]	88.74 [±25.24]	109.9 [±20.36]	148.4 [±28.41]
	1.47 [±0.56]	28.12 [±13.75]	0.361 [±0.284]	0.315 [±0.29]	0.11 [±0.155]	0.073 [±0.122]	90.82 [±32.10]	121.0 [±24.02]	139.5 [±31.60]
**S_Activation_**	1.32 [±0.29]	25.88 [±9.54]	0.241 [±0.178]	0.149 [±0.13]	0.073 [±0.067]	0.042 [±0.037]	77.17 [±29.29]	100.5 [±28.28]	120.1 [±32.54]
	1.18 [±0.39]	23.57 [±11.26]	0.283 [±0.195]	0.198 [±0.16]	0.069 [±0.067]	0.036 [±0.037]	78.35 [±40.48]	101.7 [±40.05]	125.5 [±43.82]
**Control**	1.30 [±0.39]	23.30 [±10.56]	0.279 [±0.169]	0.204 [±0.15]	0.062 [±0.071]	0.040 [±0.052]	86.21 [±20.44]	109.7 [±23.65]	137.7 [±48.99]
	1.28 [±0.44]	23.95 [±12.98]	0.377 [±0.156]	0.296 [±0.15]	0.129 [±0.145]	0.085 [±0.10]	79.19 [±25.45]	108.5 [±29.79]	123.3 [±22.40]

Data given in mean and standard deviation in parentheses before and after the intervention (amplitudes in mV; integrals in mV*ms; SICI and ICF as their index; CSP in s).

MEP: motor evoked potential. ICF: intracortical facilitation. SICI: short intracortical inhibition. CSP: cortical silent period. SL: somatosensory learning. S: somatosensory.

Similar models for ICF and CSP showed no significant interactions (p>0.05 for all models). Though there was no interaction, an exploratory analysis for CSP showed a trend for an increase in CSP in the sensory tasks only (p = 0.079; before: 101 ms ±25; after: 106±33) when comparing against baseline.

In terms of the analysis to compare differences across sensory tasks, there was no significant differences for all the outcomes of cortical excitability when comparing active tasks only; confirming that effects were similar between active tasks compared to control. Pearson’s correlation did not show any correlation between motor cortical excitability changes and altered pain thresholds.

## Discussion

This study demonstrated that sensory behavioral tasks induced laterality specific effects as they increased pressure pain thresholds of the ipsilateral left hand - thus decreasing perception of pain – and, in contrast, they had the opposite effect in the contralateral right hand, hence, increasing perception of pain. Additionally, TMS measurements showed that somatosensory input (SSI) generally decreased motor cortex excitability over time indexed by significantly reduced amplitudes of MEP and a trend for increased SICI. Interestingly, although the three sensory tasks similarly impacted pain thresholds and motor cortical excitability, only the task with visual feedback showed significant somatosensory learning (SSL). This evidence together with the lack of correlation between motor cortex plasticity and pain thresholds suggests that the effects of SSI on pain threshold were independent of motor cortical neural mechanisms.

One potential limitation of this investigation was that the assessment to measure pain (pressure pain threshold) does not measure cutaneous pain receptors only as it also measures deep muscular pain. The pressure pain threshold test rather evaluates the combined pain threshold of cutaneous pain receptors and also deeper pain receptors such as from the periost and muscular receptors [30], [31], [32], [33]. Although this may be interpreted as a limitation, our goal was to use an outcome measure that could differentiate at least at some extent the assessment of pain and the intervention tasks consisting of light sensory touch. This is why we utilized pressure pain threshold rather then a cutaneous pain assessment to assess pain. However, different effects then those we revealed could be possible using cutaneous assessments.

Nevertheless, in our experiment, somatosensory tasks changed pressure pain threshold. This is in line with results from other studies using other types of somatosensory stimulation [34], [35], thus provide further evidence that sensory stimulation can change pain perception. For instance, for the treatment of pain it was found that prosthesis with sensory feedback as well as transcutaneous electrical nerve stimulation (TENS) reduced pain [34], [35], [36]. In the present study, we tested pure SSI without any motor involvement as visually controlled and monitored by an experimenter. Since some previous studies testing somatosensory training had also a motor component that could explain effects on pain, we aimed to isolate the somatosensory involvement. We discuss then potential mechanisms to explain the sensory effects on pain threshold based on the main results we found and summarized here: (i) the effects were specific for location of stimulation (hand-specific); (ii) effects did not depend on the somatosensory task (learning-based sensory task was not different than simple sensory activation task); (iii) there was a significant decrease in motor cortical excitability.

Initially, we discuss the mechanistic insights with our first finding: *somatosensory tasks led to an increase in pain threshold in the left trained hand and an opposite effect on the untrained hand.* This finding supports the clear notion that somatosensory tasks have a hand specific effect on pain thresholds. Since, somatosensory peripheral stimulation leads to broad activation of bi-hemispheric structures [37], it might be possible that the modulation induced by the sensory tasks leads to a similar neuronal activation profile. Based on bilateral activation with sensory stimulation, it is likely that neural mechanisms besides cortical structures may explain this laterality specific effect (for instance subcortical structures such as thalamic nuclei). Supporting this conclusions are the results of a recent study in monkeys reporting that ipsilateral tactile stimuli resulted in reduced responses to stimuli on the contralateral hand (and increased in the ipsilateral hand) [38].

Our second finding strengthens the hypothesis of the involvement of non-cortical neural mechanisms as here we showed that a learning based somatosensory task had the same effect on pain processing as a simple somatosensory task. To confirm cortical involvement of the learning task, we have shown that a significant learning occurred with the sighted task. This can be explained by neuronal processing on a cortical level, since it demanded a higher level of cortical activation and processing. As all tasks led to similar effects in pain alleviation of the ipsilateral left hand and pain sensitization of the contralateral right hand, this suggest that a simple level of sensory stimulation is the only requisite to modulate pain-related neural circuits; thus subcortical structures are likely responsible for this effect. Indeed, the somatosensory learning task was likely associated with significant cortical activation as indicated by a fMRI study in which combined visual and somatosensory input increased blood oxygenation level in certain cortical areas compared to only one of the stimuli [39].

The third finding that supports our proposed mechanism of subcortical involvement is the lack of correlation between pain measurements and cortical plasticity as measured by TMS. Indeed, we found a decrease in motor cortical excitability as indexed by significantly reduced amplitudes of MEP and a trend for increased SICI. This result by itself was unexpected since we hypothesized that sensory tasks would induce increased excitability of the motor cortex [40]. This potentially conflicting result suggests that motor cortical excitability changes in our study as indexed by TMS might be an epiphenomenon and not the cause of the changes in pain threshold [41]. Furthermore, since only MEP was changed and no other intra-**cortical** measurement revealed a significant change, this provides further evidence that changes in M1 are due to secondary, non-cortical effects. In line with our findings, a study using electrical stimulation of the median nerve and digital nerves of fingers showed that motor cortical excitability decreases [42]. Nevertheless, the limitation in comparability needs to be mentioned as the study assessed changes in motor cortical excitability only immediately after stimulation. Finally we did not find a significant correlation between the decrease in cortical excitability in M1 and pain perception changes, strengthening the notion that analgesic effects due to the sensory behavioral tasks in the present study are not mediated through M1.

In conclusion, our experiment showed that sensory behavioral tasks decreased pain perception of the ipsilateral hand and increased it in the contralateral hand. Based on the hand specific effects, the lack of differences between the learning-based task and the other somatosensory tasks, and results showing a reduction of cortical excitability in the motor cortex along with no correlation with pain thresholds, it is likely that these effects on pain threshold are independent from the motor cortex and its increased excitability. It might be possible that subcortical mechanisms (potentially at thalamic or spinal cord level) are mediating these effects. Future research should then use other methods to localize specific subcortical targets associated with potential analgesic effects of somatosensory modulation as well as test these effects in patients with pain and potentially combine with top down techniques such as brain stimulation.
